# Predictors of preterm birth in Western Ethiopia: A case control study

**DOI:** 10.1371/journal.pone.0247927

**Published:** 2021-04-07

**Authors:** Misganu Teshoma Regasa, Leta Hinkosa, Merga Besho, Tilahun Bekele, Tariku Tesfaye Bekuma, Reta Tsegaye, Getahun Fetensa Hirko, Jote Markos, Aga Wakgari

**Affiliations:** 1 Department of Midwifery, Institute of Health Sciences, Wollega University, Nekemte, Ethiopia; 2 Department of Public Health, Institute of Health Sciences, Wollega University, Nekemte, Ethiopia; 3 Department of Nursing, Institute of Health Sciences, Wollega University, Nekemte, Ethiopia; 4 School of Medicine, Institute of Health sciences,Wollega University, Nekemte, Ethiopia; University of Mississippi Medical Center, UNITED STATES

## Abstract

**Background:**

Early neonatal death caused by preterm birth contributes the most for perinatal death. The prevalence of preterm birth continues to rise and is a significant public health problem. The exact cause of preterm birth is yet unanswered, as mostly preterm birth happens spontaneously. Predictors of preterm birth in developing countries like Ethiopia were not well investigated, and no study was conducted before this in the study area.

**Objectives:**

To identify predictors of preterm birth in Western Ethiopia, 2017/2018.

**Methods:**

Health facility-based unmatched case-control study was conducted from October 20/2017-march 20/2018 in 4 Hospitals. A total sample size of 358 women was recruited. From this 72 were cases and 286 were controls. Cases were mothers who gave Preterm birth, and controls were mothers who gave birth at term. Ethical clearance was obtained from Wollega University ethical review committee. A pre tested, structured questionnaire was used to collect data. Data entry and analysis was done using Epi Data 3.1 and SPSS version 21, respectively. Logistic regression was done to identify predictors of preterm birth.

**Result:**

Three hundred fifty-eight women participated in this study of which 72 were cases and 286 were controls; making the overall response rate of 100%. Lack of antenatal care visit [AOR = 3.18, 95% CI 1.37–7.38]),(Having 1–2 antenatal care visit [AOR = 2.27, 95% CI 1.18–4.35]),history of previous preterm)[AOR = 5.19, 95% CI1.29–20.88],Short Interpregnancy Interval [AOR = 4.41.95% CI 2.05–9.47],Having Reproductive tract infections [AOR = 2.54, 95% CI 1.02–6.32] and having Obstetric complications [AOR = 2.48,95% CI 1.31–4.71] were found to be predictors of preterm birth.

**Conclusion and recommendation:**

Risk factors of preterm delivery are multifactorial and depend on geographical and demographic features of the population studied. Hence results of studies from one area might not be applicable to another area. Antenatal care visits are unique opportunities for early diagnosis and treatment of problems. Therefore, antenatal care should be strengthened, and appropriate counseling should be given at each antenatal care follow up. Maintainning optimum birth interval through family planning, and early identification and treatment of reproductive tract infections are mandatory.

## Introduction

World Health Organization (WHO) defines preterm birth as all birth less than 37 completed weeks of gestational age or fewer than 259 days since the first day of last normal menstrual period. Fifteen million preterms happen each year globally and remain the major contributor of neonatal morbidity and mortality. [1–3]. There is substantial progress in preterm babies’ care, yet preterm birth prevalence is on the rise and remains a global challenge in developing and developed countries [2–4].

Preterm birth is the single most common causes of perinatal mortality in developing and developed countries; its adverse health outcomes even worse in developing countries [5–8]. Moreover, there is a high parity and infectious disease like anemia and malaria in developing countries, which may confound the association between predictors of preterm birth [7–9]. Studies indicated maternal parity, short interpregnancy interval, low socioeconomic state, emotional stress, lack of regular antenatal care, and antepartum hemorrhage as predictors of preterm birth [10–12].

Case—control study conducted in Yemen indicated that women who gave birth of premature infants were less educated (60% vs 53%), more socioeconomically deprived (67% vs 50%), and rural residence (65% vs 60%) irrespective of birth spacing. Maternal age <20 years was observed in high proportion (43.2%) among cases with IPI <12 months compared to 16.6% in controls [11–15]. Across-sectional study conducted in Felegehiwot Referral Hospital indicated that Preterm delivery was higher 14(66.7) in rural residence [16].

Early neonatal death caused by preterm birth contributes the most for perinatal death. Even if they were delivered preterm, infants could survive. However, they often face lifelong adverse health effects such as respiratory difficulties, cerebral palsy, vision and hearing loss, feeding and digestive problems, and intellectual disabilities [17–19]. The exact cause of preterm birth is still unanswered, as mostly preterm birth happens spontaneously [8]. At the same time the etiology of preterm birth is multi factorial and it is affected by sociodemographic characteristics of the population under the study [20, 21].

Therefore, more investigation is needed to identify preterm birth predictors for effective intervention strategies [8]. Risk factors of preterm delivery in developing countries particularly in Ethiopia was not well investigated, and no prior study was conducted in the study area. Therefore this is study aimed to assess predictors of preterm birth in western Ethiopia.

## Methods

### Study area and period

This study was conducted in East Wollega, West Wollega, Horo Guduru Wollega and Kellem Wollega zones of Oromia, Western Ethiopia. The study was conducted from October 20/2017-march 20/2018.

### Study design

Hospital-based unmatched case control study was conducted.

### Population

#### Source population

All mothers who gave birth after 28 weeks of gestational ages in selected hospitals.

#### Study population

Mothers who gave birth after 28 weeks of gestational age in the selected hospitals’ during data collection period.

### Eligibility criteria

#### Inclusion criteria

Mothers with singleton birth were included in the study.

#### Exclusion criteria

Those who had an abortion in the preceding pregnancy (since mothers who had abortion have recommended interpregnancy interval of 6 months).

#### Sample size and sampling techniques

A sample size of 72 cases and 286 controls was determined using the methods of "difference between population proportions" with 80% power, 95% confidence level. The ratio of cases to controls being one to four… The proportion of short interpregnancy interval among controls considering 46% [15] and taking odds ratio of 2.2 for interpregnancy interval of <24 months [22]. The non-response rate is estimated to be 5%, and hence an overall sample size of 358 women was recruited in the study. Four hospitals found in four zones of Wollega were included in this study. The number of subjects was selected from each hospital based on the cases load during the study period. All mothers who fulfil the inclusion criteria and give birth to preterm were included as cases. Four controls were selected for every case identified from the same institution. The classification of cases and controls was based on preterm babies born from those mothers in the four hospitals. Those mothers who are given birth of preterm [17] <37 Weeks was designated as cases, and those who are given term babies [37–42 weeks] were designated as controls.

#### Data collection procedure

Data was collected by face to face interview using a structured and pretested questionnaire first prepared in english for this study and translated to Afan Oromo (the official language of the region) before the start of the fieldwork. Data was collected by 12 B.Sc midwives who are fluent in the official language of the region from October 20/2017-March 20/2018. First, data collectors identified mothers who had given birth to a preterm baby (**cases**) and who have not (**controls**). Then, they interviewed both groups to determine their exposure status. Thus, four mothers who had given term babies were interviewed for every mother who gave birth to a preterm baby. The supervisor was assigned to supervise the data collection process and perform quality checks.

#### Data quality control

The quality of data was assured by proper designing and pretesting the questionnaires in Nedjo Hospital on 5%. Both the data collectors and supervisors were given two days comprehensive training before the actual work about the aim of the study, procedures, data collection techniques, art of interviewing going through the questionnaire question by question.

To ensure the questions are cear and clear understandable by both the interviewers and the respondents, the questionnaire was pretested and further refined based on the results. Every day after data collection, questionnaires were reviewed and checked for completeness by the supervisors and the necessary feedback were offered to data collectors in the next morning. The issue of confidentiality and privacy was focused during the training session, and data collectors participated in pretesting the questionnaire after training.

### Study variables

➢ **Dependent /Outcome variable**
                      Preterm birth
➢ **Independent variables**

Sociodemographic characteristics: Maternal age, marital status, level of Education, occupation, smoking

Obstetric Characteristics: Parity, Gravidity, ANC attendance, Number of ANC visits, IPI. Medical problems (HIV Infection, PIH)

### Operational definition

#### Preterm birth

A birth which occurs within less than 37 completed weeks

#### Cases

Mothers who gave birth to preterm babies during data collection period in the selected hospitals.

#### Controls

Mothers who gave birth to term babies during data collection period in the same selected hospitals.

#### Inter-pregnancy interval

Interval between delivery of a live birth of previous pregnancy and the birth of the current child Which is calculated as the interval between the birth of the most recent previous child and the birth of the current child minus the gestational age of the current child.

#### Data processing and analysis

The data gathered through the structured questionnaire was, compiled, Cleaned, coded and entered in to EpiData version 3.1 and exported to SPSS version 20 for analysis. Frequency was used to clean data. All the variables were entered in to bivariate analysis and those explanatory variables with a p value < 0.2 in crude analysis were considered as a candidate variable for multivariable analysis and those variables with a p value < 0.05 in multivariable analysis were considered as significant association. Strength of association was presented using odds ratios with 95% confidence intervals.

## Ethical considerations

Ethical clearance was obtained from Wollega University College of Health Science Institutional Research Ethics Review Committee (IRERC). Written informed consent was obtained from each participant after fully explaining the study’s purposes before the interview. Formal letter of cooperation was written for Each respected zonal Health Office and respective Hospitals. Participants are informed about the study’s objective and their rights that it contributed necessary information for policy makers and other concerned bodies. Any involvement in the study was after their full consent is obtained. They were also informed that all data obtained from them would be kept confidential by using codes instead of any personal identifiers and is meant only for the purpose of the study. The interviews were conducted in separate private places.

## Result

### Sociodemographic characteristics of cases (n = 72) and Controls (n = 286) in selected Hospitals of Wollega zones, 2017/18

Three hundred fifty-eight women participated in this study of which 72 were cases, and 286 were controls; making the overall response rate of 100% [Table 1]

**Table 1:** Sociodemographic characteristics of cases (n = 72) and controls (n = 286) in selected hospitals of Wollega zones, 2017/18.

Variables	Control n [%]	Cases n [%]
	[n = 286]	[n = 72]
**Age**		
< = 20	31[10.8]	10[13.9]
21–34	237[82.9]	53[73.6]
>34	18[6.3]	9[12.5]
**Religion**		
protestant	187[65.4]	47[65.3]
Orthodox	64[22.4]	20[27.8]
Muslim	26[9.1]	3[4.2]
Wakefata	9[3.1]	2[2.8]
**Ethnicity**		
Oromo	242[84.6]	56[77.8]
Amhara	30[10.5]	13[18.1]
Gurage	14[4.9]	3[4.7]
**Residency**		
Urban	157[54.9]	39[54.2]
Rural	129[45.1]	33[45.8]
**Occupational status**		
House wife	135[42.2]	36[50]
Government Employee	71[24.8]	16[22.2]
Merchant	60[21]	12[16.7]
Daily laborer	20[7]	8[11.1]
**Educational Status**		
Unable to read and write	64[22.4]	14[19.4]
Elementary	72[25.2]	26[36.1]
Secondary	88[30.8]	18[25]
College and above	62[21.7]	14[19.4]

## Predictors of preterm birth

In the bivariate analysis, number of ANC Visit, history of abortion, sex of the newborn,ANC follow up, Iron supplementation, outcome of the previous delivery, interpreganancy interval, reproductive tract infection, obstetric complication and anxiety during pregnancy were found to be predictors of preterm birth. multiple logistic regressions showed that the number of ANC visit, the outcome of the previous delivery, interpregnancy interval, reproductive tract infections, and obstetric complications were predictors of preterm birth, revealing a significant association with preterm birth in this study [Table 2]. Compared to those with 3–4 ANC visits, women with 1–2 ANC visits and those with no visit had 2.7 (AOR = 2.27,95%CI 1.18–4.35) and 3.2 (AOR = 3.2,95%CI 1.37–7.38) times higher risk to have a preterm birth, respectively.

**Table 2:** Binary and multivariable logistic analysis of predictors of preterm birth cases (n = 72) and controls (n = 286) in selected hospitals of Wollega zones, 2017/18.

Variables	Controln[%],	Cases n [%]	COR[95% CI]	AOR[95% CI]
	[n = 286]	[n = 72]		
**Number of ANC**				
**None**	27[62.8]	16[37.2]	4.56[2.14–9.71] *	3.18[1.37–7.38]*
**1–2**	82[71.3]	33[28.7]	3.09[1.71–5.6] *	2.27[1.18–4.35]*
**3–4**	177[88.5]	23[11.5]	1	1
**History of Abortion**				
**Yes**	39[67.2]	19[32.8]	2.27[1.22–4.24]*	1.49[0.68–3.28]
**No**	247[82.3]	53[17.7]	1	1
**Sex of the Newborn**				
**Male**	135[74.6]	46[25.4]	1.98[1.16–3.37]*	1.72[0.94–3.27]
**Female**	151[85.3]	26[14.7]	1	1
**ANC Follow up**				
**Yes**	259[82.2]	56[17.8]	1	1
**No**	27[62.8]	16[37.2]	2.74[1.38–5.42]*	2.43[0.96–6.18]
**Iron Supplementation**				
**Not taken**	41[81.2]	26[38.8]	4.06[1.41–11.75]*	2.30[0.66–8.03]
**< = 3month**	213[83.9]	41[16.1]	1.23[0.45–3.35]	1.12[0.36–3.48]
**>3month**	32[86.5]	5[13.5]	1	1
**Outcome of the previous delivery**				
**Prim**	57[80.3]	14[19.7]	1	1
**Term alive**	194[85.1]	34[14.9]	0.71[0.36–1.42]	0.75[0.37–1.52]
**Term stillbirth**	20[69.0]	9[31.0]	1.83[0.69–4.88]	1.31[0.49–3.96]
**Preterm**	4[33.3]	8[66.7]	8.14[2.14–30.94]*	5.19[1.29–20.88]*
**Abortion**	11[61.1]	7[38.9]	2.59[0.85–7.89]	1.83[0.56–6.00]
**IPI**				
**Prim**	48[87.3]	12.7[12.7]	O.74[0.21–1.84]	0.79[0.30–2.07]
**<18monts**	24[47.1]	27[52.9]	5.68[2.28–11.60]*	4.41[2.05–9.47]
**18-23months**	103[86.6]	16[13.4]	0.78[0.39–1.57]	1.02[0.49–2.15]
**>23months**	111[83.5]	22[16.5]	1	1
**RTI**				
**Yes**	13[56.5]	10[43.5]	3.39[1.42–8.07]*	2.54[1.02–6.32]*
**No**	273[81.5]	62[18.5]	1	1
Faced obstetric complication**				
**Yes**	82[64.6]	45[35.4]	4.15[2.41–7.13]	2.48[1.31–4.71]
**No**	204[88.3]	27[11.7]	1	1
**Anxiety during pregnancy**				
**Yes**	15[62.5]	9[37.5]	2.58[1.08–6.17] *	1.86[0.63–5.50]
**No**	271[81.1]	63[18.9]	1	1

**1**Reference

*Significant association at P value <0.05

**Face obstetric complication:APH,Passage of liquor,PIH,GDM, Oligohydramnious and Polyhydramnious.

Similarly, Compared to those with no history of previous preterm birth; those who had a history of previous preterm birth were 5.19 times the high risk of preterm birth [AOR = 5.19, 95% CI1.29–20.88]. Likewise, those Women giving preterm birth were 4.41 more likely to have an inter-pregnancy interval of less than 18 months than those giving birth at term [AOR = 4.41.95% CI 2.05 9.47]. Similarly, those Women giving preterm birth were 2.54 more likely to have reproductive tract infection than those giving birth at term [AOR = 2.54, 95% CI 1.02–6.32]. Correspondingly, those women who gave preterm birth were 2.48 more likely to have pregnancy complication than those giving birth at term [AOR = 2.48, 95% CI 1.31–4.71] [Table 2].

## Discussion

This study identified the number of ANC visits, the outcome of previous delivery (previous history of preterm birth), short interpregnancy interval, having reproductive tract infection, and having pregnancy complications as a predictor of preterm birth. Compared to those with 3–4 ANC visits, women with 1–2 ANC visits and those with no ANC visit had 2.7 (AOR = 2.27,95%CI 1.18–4.35) and 3.2 (AOR = 3.2,95%CI 1.37–7.38) times higher risk to have a preterm birth, respectively.

This is similar to the previous study conducted at Qom Hospitals, Yemen, and a cross-sectional study conducted at Debremarkos Ethiopia [11, 15, 23]. This could be because women with regular ANC follow-up may have more chance to detect and treat obstetric complications. Therefore, enhancing and strengthening the ANC services plays a vital role in reducing preterm birth.

Similarly, those women giving preterm birth were 5.19 more likely to have a history of previous preterm birth than those giving birth at term [AOR = 5.19, 95% CI1.29–20.88]. This finding is consistent with a previous cross-sectional study in Ethiopia at Felegehiwot referral hospital, Debremarkos town, and Western China [17, 23, 24]. This may be due to the tenacity of unknown risk factors of the previous delivery. Therefore, a woman with prior preterm birth may benefit from increased clinical attention and assessment to identify risks and provide appropriate interventions for the prevention of preterm birth.

Women giving preterm birth were 4.41 more likely to have an inter-pregnancy interval of fewer than 18 months than those giving birth at term [AOR = 4.41.95% CI 2.05–9.47]. This is in line with a Case-control study conducted in Yemen, Qom Hospitals, Scotland, and Brazil [9, 11, 15, 16], and in contrast with the study conducted in Boston (USA) and Cross-sectional study conducted in Addis Ababa [6, 13]. This difference could be due to the difference in geographical and demographic features, the methods difference, and lack of controlling confounders like the population studied socioeconomic status. Thus, encouraging and reassuring the recommended inter-pregnancy interval will reduce the prevalence of preterm birth. Similarly, those Women giving preterm birth were 2.54 more likely to have reproductive tract infection than those giving birth at term [AOR = 2.54, 95% CI 1.02–6.32]. So, early identification and treatment of STIs/RTIs are as important during pregnancy as at any other time in reducing preterm birth.

Women who gave preterm birth were 2.48 more likely to have pregnancy complications than those giving birth at term [AOR = 2.48,95% CI 1.31–4.71]. This is in line with the cross-sectional study conducted in the Debre markos town and the case-control study conducted in Southern India [23, 25]. These could be due to most of the pregnancy complications affect the placental function like delivery of oxygen and nutrients to the growing fetus, possibly resulting in fetal growth restriction. Furthermore, medical problems during pregnancy can increase the risk of preeclampsia/Eclampsia and increase the risk of preterm birth.

## Limitations of the study

As this is a case-control study, it is not as powerful as other studies confirming a causal relationship. Case-control studies are often used to provide early clues and inform further research using more rigorous scientific methods. Since the survey deals with sensitive issues, there is a possibility of falsified reporting among mothers, especially given the face-to-face interview modality of data collection. Another limitation of the study is being institution based study; the number of institutional delivery is very low in Ethiopia and this study may not represent the general population.

## Conclusion

The etiology of preterm birth is multifactorial and depends on the population studied geographical and demographic features; hence, the results of studies from one area might not apply to another site. This study identified that number of ANC visit, previous history of preterm birth, short interpregnancy interval, having reproductive tract infection and having pregnancy complication as a predictor of preterm birth.

### Recommendation

ANC visits are unique opportunities for early diagnosis and treatment of problems and play an important role in the women’s reproductive health, mainly through caring for pregnant women and early identification and treatment of obstetric complications. Therefore, ANC visits should be strengthened, and appropriate counseling should be provided at each visit of ANC to avoid missed opportunities and address reproductive health needs, especially family planning to promote optimum birth spacing. Even though there is no Strict optimal IPI has not been agreed upon universally, <18 months’ interval was a period found to be associated with a high risk of preterm birth. The women with a previous history of preterm birth were also in a high-risk group whose health professionals should pay more attention during antenatal care. Similarly, early identification and treatment of RTI are also crucial.

## Supporting information

S1 Questionnaires (DOCX)

S2 Questionnaires (DOCX)

S1 Data (SAV)

## Abbreviations

ANC: Antenatal care
FMOH: Federal Ministry of Health
HIV: Human Immuno Virus
IPI: Interpregnancy Interval
IRERC: Institutional Research Ethics Review Committee
PIH: Pregnancy Induced Hypertension
PROM: Premature rupture of membrane
RTI: Reproductive tract infection
WHO: World health Organization
