# Supplementary material for: Predictors of preterm birth in Western Ethiopia: A case control study
Source: PLoS One. 2021 Apr 7;16(4):e0247927. doi: 10.1371/journal.pone.0247927 (PMC8026033; doi:10.1371/journal.pone.0247927)
Supplement: S1 Questionnaires — (DOCX) [file pone.0247927.s001.docx]

Questionnaires to **assess** **Predictors of Preterm birth in Western Ethiopia: A Case Control Study.**

If Respondent agrees to be interviewed: starting time___________: End time_________:

001. Questionnaire Code __________

Date of data collection________/_________/_________

Name of data collector__________________________ signature_______________

Name of supervisor_____________________________ signature_______________

**Part one:** Sociodemographic Characteristics

| **Sr.no** | **Questions** | **Choice answers** | **Skip to ___** |
| --- | --- | --- | --- |
| 101 | Mothers Age | in years**_______** |  |
| 102 | Residence | 1.Urban  2.Rural |  |
| 103 | Marital Status: | 1. Married  2. Divorced  3. Widowed  4. single |  |
| 104 | Religion | 1.Protestant  2.Orthodox  3.Muslim  4.others, specify_______ |  |
| 106 | Ethnicity | 1. Oromo  2. Amhara  3.Gurage  4. Others, specify_____ |  |
| 107 | occupation(mother) | 1. House wife  2. Gov’t Employee  3. farmer  4. merchant  5. daily labourer  6.Others Specify______ |  |
| 108 | Educational status of the mother | 1. No education  2. Primary  3. Secondary  4 .tertiary and Above |  |
| 109 | Average monthly family income | in birr_________ |  |

**Part 2:** Obstetric characteristics

| **Sr.no** | **Questions** | **Choice answers** | **if no Skip to** |
| --- | --- | --- | --- |
| 201 | Gravidity | **___________** |  |
| 202 | Parity | **___________** |  |
| 203 | When was your LMP? (Dd/mm/yr) | ______/______/_______ |  |
| 204 | Date of deliveries? (Dd/mm/yr | ______/______/________ |  |
| 205 | Gestation by dates in weeks | _______in weeks |  |
| 206 | Outcome of delivery | 1.preterm  2.Term |  |
| 207 | Sex of the baby | 1.Male  2.female |  |
| 208 | Have you attended ANC follow up when you were pregnant? | 1.Yes  2.No | **If no skip to 210** |
| 209 | If yes Number of Visits | - 1. Visit   2.2 visit  3.3 visit  4.>4 visit |  |
| 2010 | HIV status | 1.Positive 2.Negative 3.Unknown status |  |
| 2011 | Supplement folic acid during pregnancy | 1. Not taken 2. <=3 Months 3. >3 Months |  |
| 2012 | How many children you have? | __________in number |  |
|  | Outcome of previous pregnancy | 1.Primi (none)  2.Term alive  3.Term still birth  4.Preterm  5.Abortion |  |
| 213 | Have you ever heard about optimal birth interval duration between two consecutive births? | 1.Yes  2.No | **If no skip to 216** |
| 214 | If yes to question no 2013, what is the optimum duration in years between two successive births? | **_______________**years |  |
| 215 | Interval between the current and previous pregnancies (months) | 1.<18 months  2.18-23 months  3.>23 months |  |
| 216 | Do you faced Obstetric complications? | 1. Antepartum hemorrhage 2. Passage of liquor 3. Pregnancy induced hypertension 4. Gestational diabetes mellitus 5. Oligohydramnios 6. Polyhydramnios |  |
| 217 | Anxiety during pregnancy | 1.yes  2.No |  |
| 218 | Reproductive tract infection during pregnancy | 1.yes  2.No |  |
