# Supplementary material for: Predictors of preterm birth in Western Ethiopia: A case control study
Source: PLoS One. 2021 Apr 7;16(4):e0247927. doi: 10.1371/journal.pone.0247927 (PMC8026033; doi:10.1371/journal.pone.0247927)
Supplement: S2 Questionnaires — (DOCX) [file pone.0247927.s002.docx]

Questionnaires to **Assess** **Predictors of Preterm birth in selected Hospitals of East, West, Horro guduru and Kellem Wollega Zones, West Ethiopia, 2017/18: A Case Control Study.**

**UNKA WAADA**

Maqaan kiyya ____________________jedhama.kanin dhuufee garee Barsiisota wallaggaa Yuunivarsitiin qorannoo geggeeffamaa jiruuf odeeffannoo funaanuuf yoo ta’u, kayyoon qorannoo Kanaas Wantoota haati tokko utuu ji’a hin guutin deessu godhan qorachuufi. Odeeffannon isin kennitan hundi iccitiidhaan qabama. Maqaa fi iddoo jireenya keessanii barreessuun hin barbaachisu. Qorannoo kana keesatti hirmaachuun fedhi irratti kan hundaa’ee ta’a. Hirmaachuu fi dhisuuf mirgi keessan kan kabajamee dha. Haata’u malee galmaan ga’umsa kaayyoo qoranichaatiifii fooya’insa tajaajila fayyaa haadholiif jecha hirmaannaan keessan murteesaa waan ta’eef akka gaaffileedhaaf deebi kennudhaan hirmaattan kabajaan isin gaafadha. Kanaaf qorannoo kana keessatti hirmaachuuf fedhii keessanii?

1.Eyyee 2. Lakki-----------yoo ta’e gara itti aanuutti darbi

Yoo Hirmaataan walii gale: Sa’a itti jalqabe___________: Sa’a itti xumurame_________:

Guyyaa Odeeffannon itti Funaaname________/_________/_________

**Maqaa Nama odeeffannoo funaanee**__________________________ Mallattoo_______________

Maqaa to’ataa_____________________________ Mallattoo_______________

**Kutaa tokkoffaa:** Gaaffiilee Aadaafi Hawaasummaa Ilaala

| **Tartiiba**  **Lakk.** | **Gaaffii** | **Filannoo deebii** | **Gara ___darbi** |
| --- | --- | --- | --- |
| 101 | Waggaankee meeqa? | Waggaadhaan**_______** |  |
| 102 | Waggaan abbaa manaakee meeqa? | Waggaadhaan ________ |  |
| 102 | Bakka Jireenyaa | 1.Magaalaa  2.Baadiyaa |  |
| 103 | Haalli gaa’eelakee akkam? | 1. Kan heerumte  2. kan hiiktee  3. Kan irraa du’e  4. Kan hin heerumne |  |
| 104 | Amantiinkee maali? | 1.pirootestaantii  2.Ortodoksii  3.Islaamaa  4.Waaqeffetaa  5.Kan biraa ibsi______ |  |
| 106 | Qomoonkee maali? | 1. Oromoo  2. Amaaraa  3.Guraagee  4. kan biraa ibsi__________ |  |
| 107 | Hojiinkee Maali? | 1. Giiftii Manaa  2. Hojjettuu Mootummaa  4. Daldaltuu  5. Hojjettuu guyyaa  6.Kan biraa ibsi______ |  |
| 108 | Sadarkaan barumsakee hagam? | 1. Kan hin baranne  2. Sadarkaa tokkoffaa  3. Sadarkaa lammaffaa  4 .Kolleejjii fi isaa ol |  |
| 109 | Galiin maatiikee ji’aan qarshii hagam ta’a? | qarshii_________ |  |
| 110 | Qabeenya Maatiikee ollaakee wajjin yoo madaaltee maal fakkaataa? | 1.Baayyee sooressa  2. Sooressa  3. Hiyyeessa  4.Baayyee Hiyyeessa |  |

**Kutaa lammaffaa:** **Gaaffiilee Da’umsa ilaalan**

| **Tartiiba**  **Lakk** | **Gaaffii** | **Filanno deebii** | **Gara__tti darbi** |
| --- | --- | --- | --- |
| 201 | Yeroo meeqa ulfoofte/**Gravidity**? | **___________** |  |
| 202 | Yeroo meeqa deessee/**Parity**? | **___________** |  |
| 203 | Marsaa laguu isa dhuma yoom argite/**LNMP?** (Dd/mm/yr | ______/______/_______ |  |
| 204 | Yoom deesse (Guyyaa da’umsaa)? (Dd/mm/yr) | ______/______/________ |  |
| 205 | Toeban meeqatti deesse/GA ? | _______in weeks |  |
| 206 | Mucaan ji’a guutee dhalateera?  (Outcome of delivery)? | 1.Ji’a hin guunne**(preterm)**  2.Ji’a guuteeraa **(Term)** |  |
| 207 | Saala mucaa dhalatee? | 1.Dhiira  2.Dhalaa |  |
| 208 | Hordoffii ulfaa godhaa turteettaa? | 1.Eeyyee  2.Lakkii | Lakkii yoo ta’e gara 210tti darbi |
| 209 | Hordoffii qabda yoo ta’e yeroo meeqa ilaalamtee? | 1. Yeroo tokko 2. Yeroo lama 3. Yeroo sadi 4. Yeroo afuriif isaa ol |  |
| 2010 | Qorannoo HIV Gooteettaa (**HIV status)** | 1.Qabdi (Positive)  2.Hin qabdu (Negative)  3.Hin beekamu (Unknown status) |  |
| 2011 | Iranii(Iron) yeroo ulfaa fudhataa turteettaa? | 1. Hin fudhanne 2. <=3 Ji’a 3. >3 Ji’a fudheera |  |
| 2012 | Ijoollee meeqa qabda? | __________Lakkoofsaan |  |
| 213 | Haati tokko deesse deebitee ulfaa’uuf yeroo hagamii turu akka qabdu dhageessee beektaa  (**Interpregnancy interval)?** | 1.Eeyyee  2.Lakkii | **Lakkii yoo ta’e gara 217 darbi** |
| 214 | Gaaffii 2013 eeyyee yoo ta’e Haatii tokko hagam walirraa fageessitee da’uu qabdi jettee yaadda? | **_______________**Waggaadhan |  |
| 215 | Mucaakee ias amma deesse kanaaf isa duraa gidduu ji’a meeqatu jira(Mucaakee duraa deessee ji’a meeqatti ulfooftee | 1 J i’a. <18  2.Ji’a 18-23  3.Ji’a >23 |  |
| 216 | Rakkoon ulfaa wajjin wal qabatu si mudatee beekaa? | 1. Da’umsa dura dhiiguu [APH] 2. Bishaan gubbee dhangala’uu 3. Dhiibbaa dhiigaa yeroo ulfaa( PIH) 4. Dhiibee sukkaaraa yeroo ulfaa [GDM] 5. Bishaan gubbee xiqqaachuu 6. Bishaan gubbeebaayyachuu |  |
| 217 | Yeroo ulfaa want si dhiphisu tureeraa? | 1.Eyyee  2.Lakkii |  |
| 218 | Qaama saal-quunnamtii keerra dhukkubbiin jiraa? | 1.Eyyee  2.Lakkii |  |
